# Supplementary material for: Synthesis, Photoluminescence and Vibrational Properties of Aziridinium Lead Halide Perovskites
Source: Molecules. 2022 Nov 17;27(22):7949. doi: 10.3390/molecules27227949 (PMC9698367; doi:10.3390/molecules27227949)
Supplement: Supplementary file 1 [file molecules-27-07949-s001.zip › molecules-2007411-supplementary.pdf]

Supporting information for

Synthesis, Photoluminescence and Vibrational Properties of Aziridinium Lead Halide Perovskites

Dagmara Stefańska*, Maciej Ptak and Mirosław Mączka*

Institute of Low Temperature and Structure Research, Polish Academy of Sciences, Okólna 2, 50-422 Wrocław, Poland

e-mail: d.stefanska@intibs.pl; m.maczka@intibs.pl

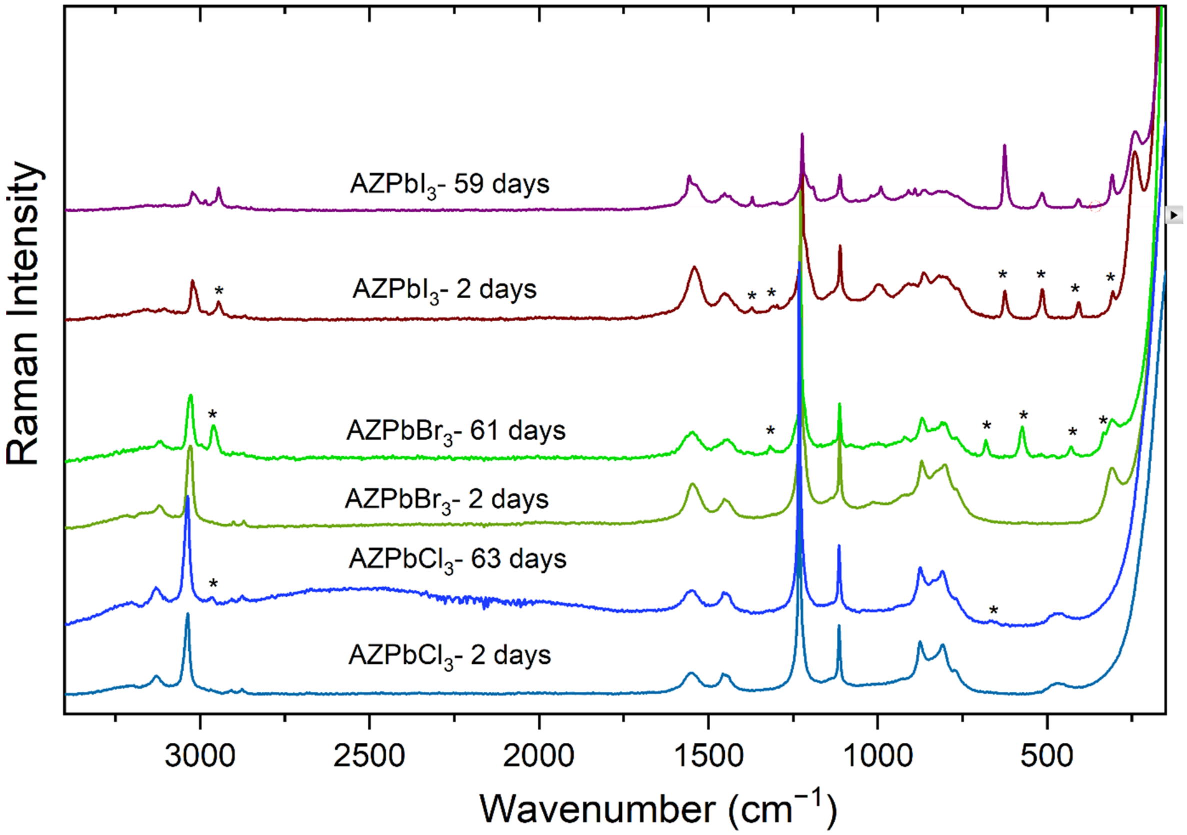

Figure S1. Raman spectra of the freshly synthesized $AZPbX_3$ samples (2 days) and the samples kept in ambient conditions for 59-62 days. Asterisks denote the bands, which appear due to presence of impurity phases.

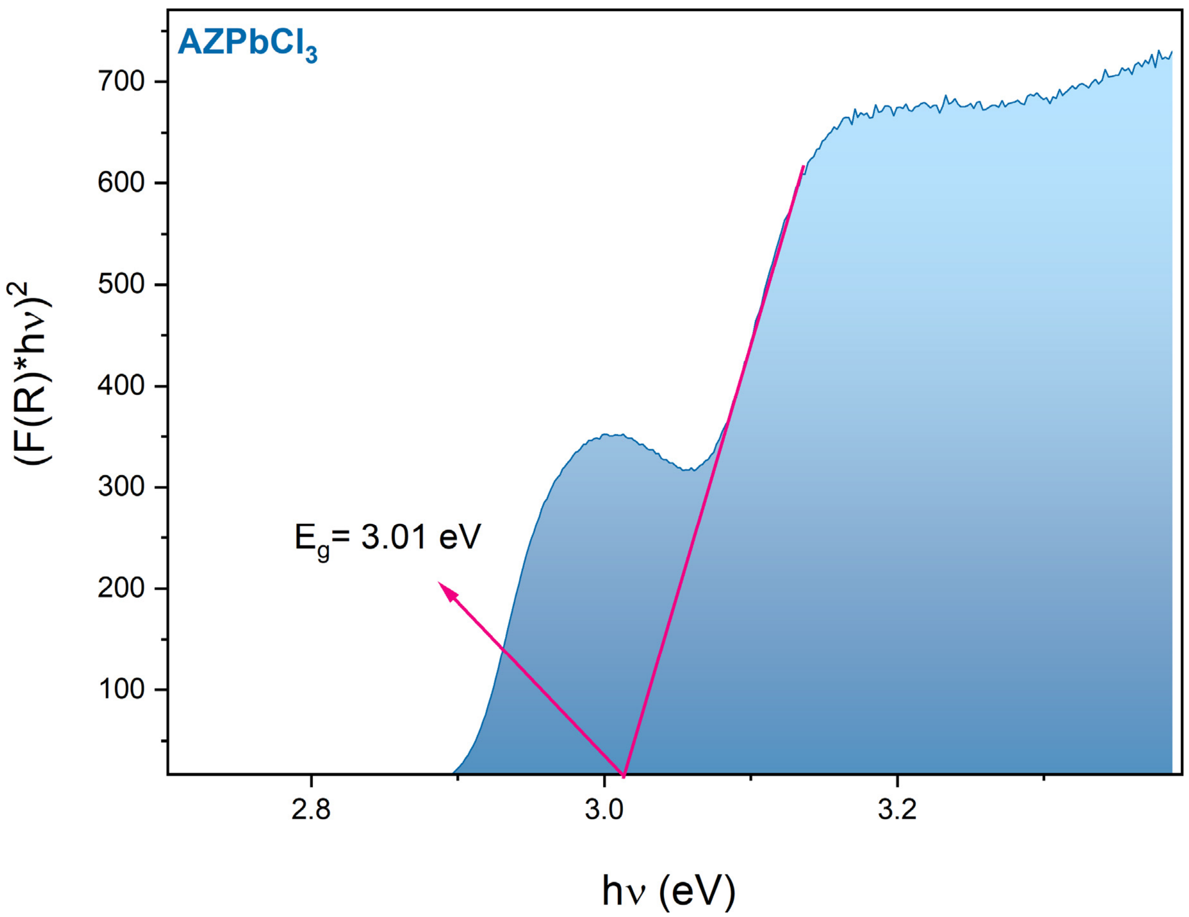

Figure S2. Energy band gap of $AZPbCl_3$ estimated using Kubelka-Munk function.

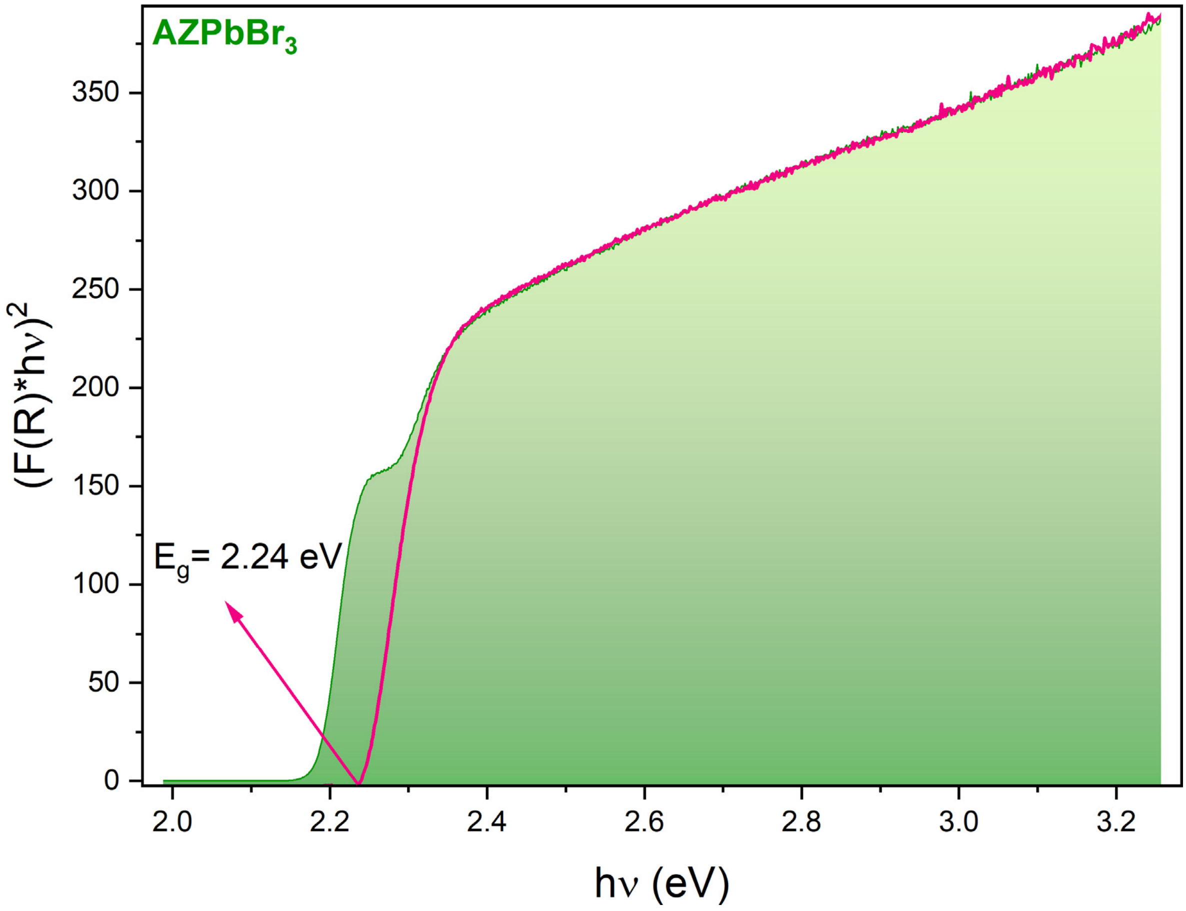

Figure S3. Energy band gap of $AZPbBr_3$ estimated using Kubelka-Munk function.

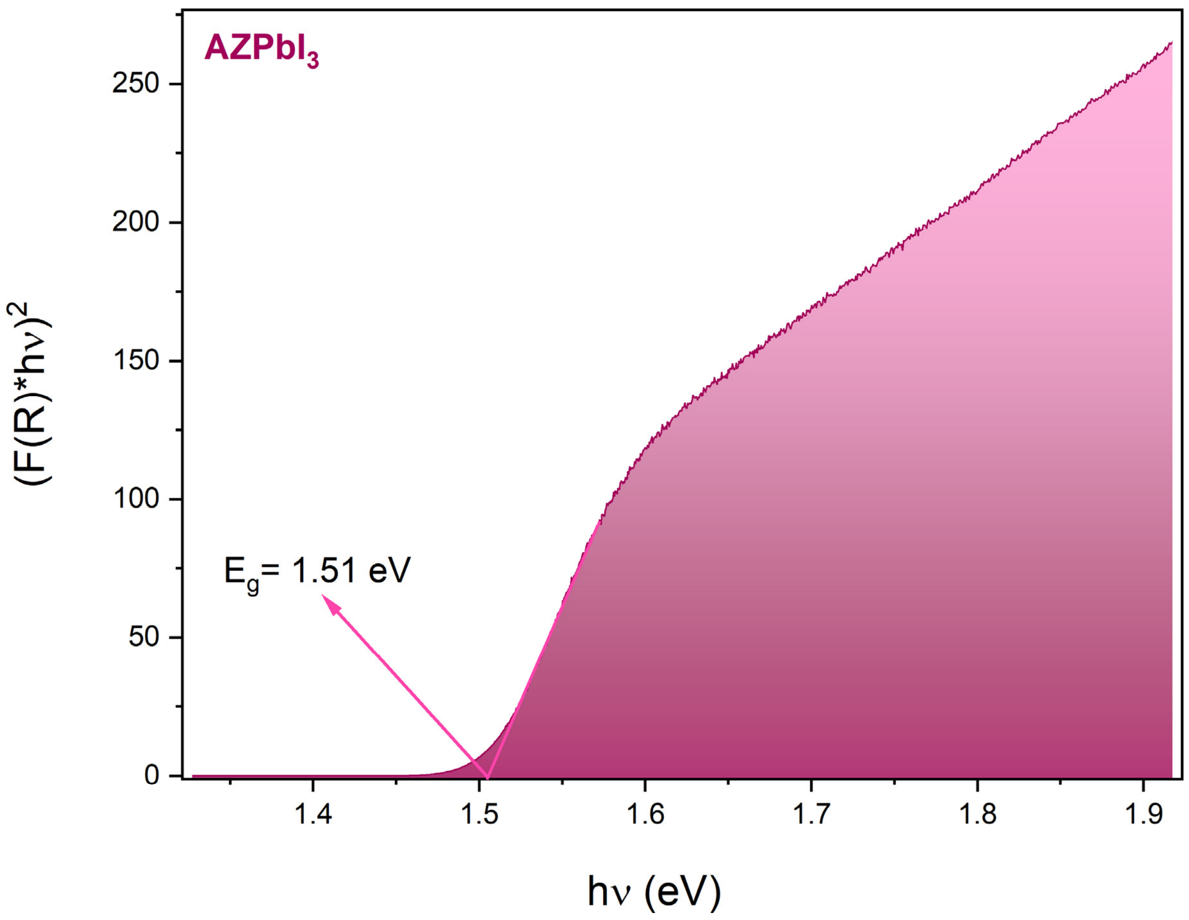

Figure S4. Energy band gap of $AZPbI_3$ estimated using Kubelka-Munk function.

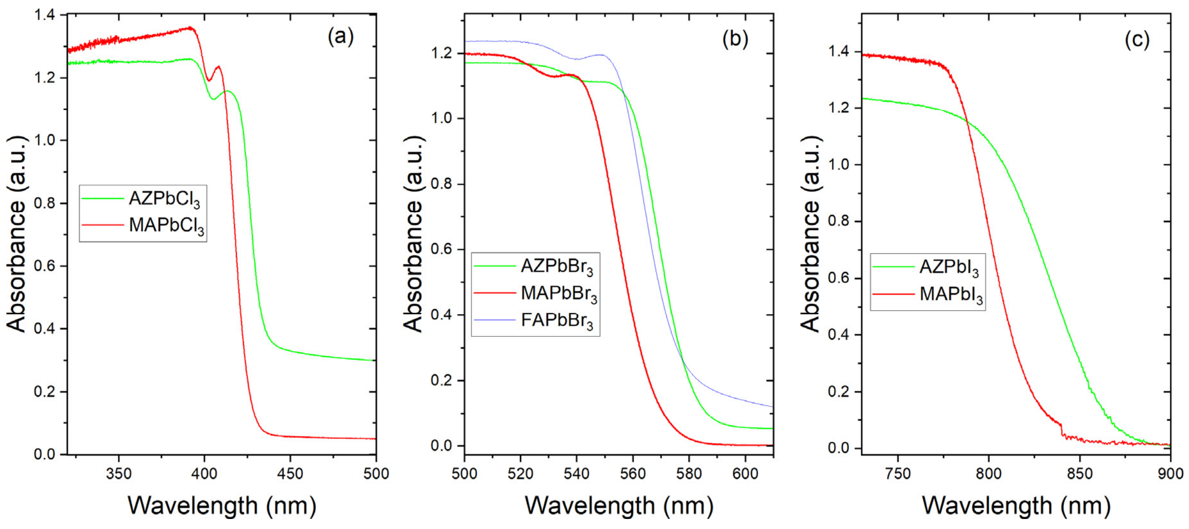

Figure S5. Diffuse reflection spectra of lead (**a**) chlorides, (**b**) bromides and (**c**) iodides.

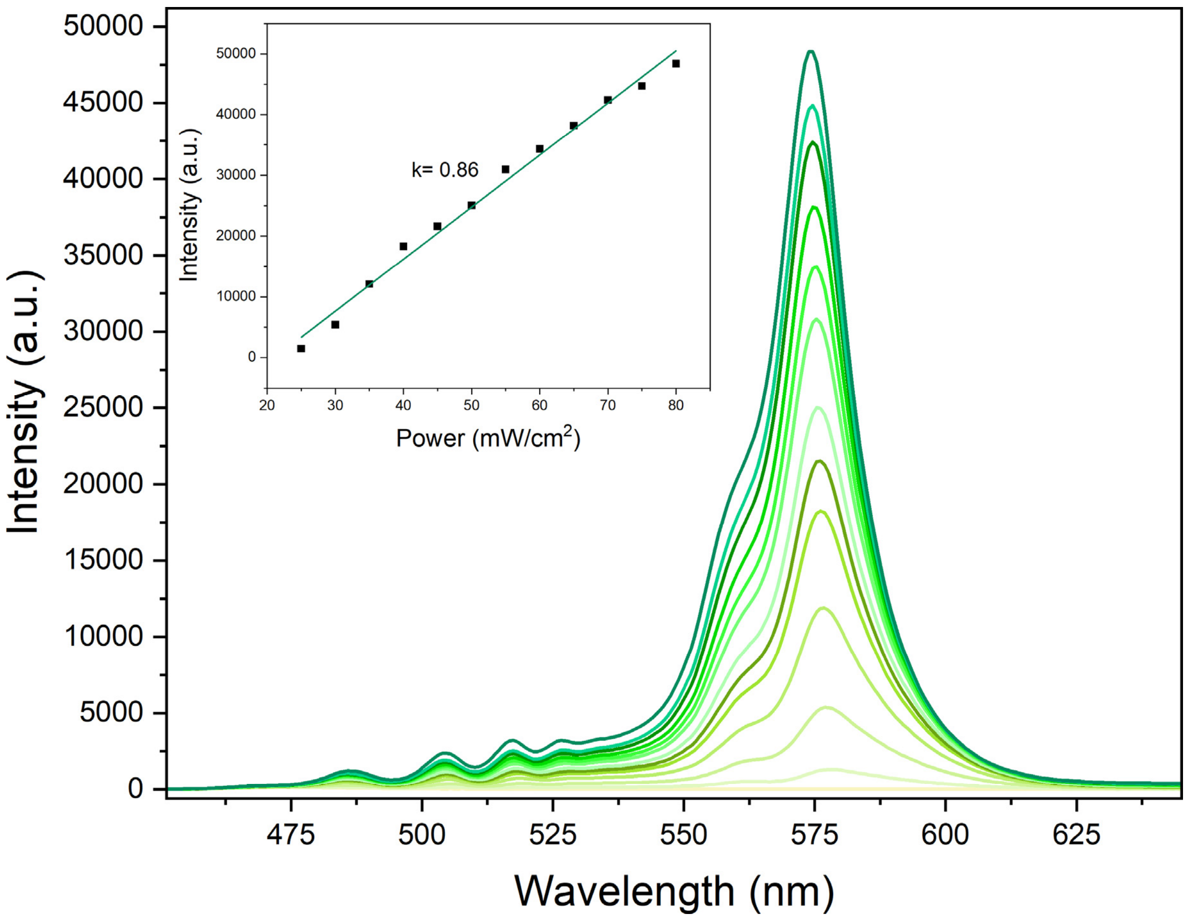

Figure S6. Dependence of PL intensity of $AZPbBr_3$ on excitation power.

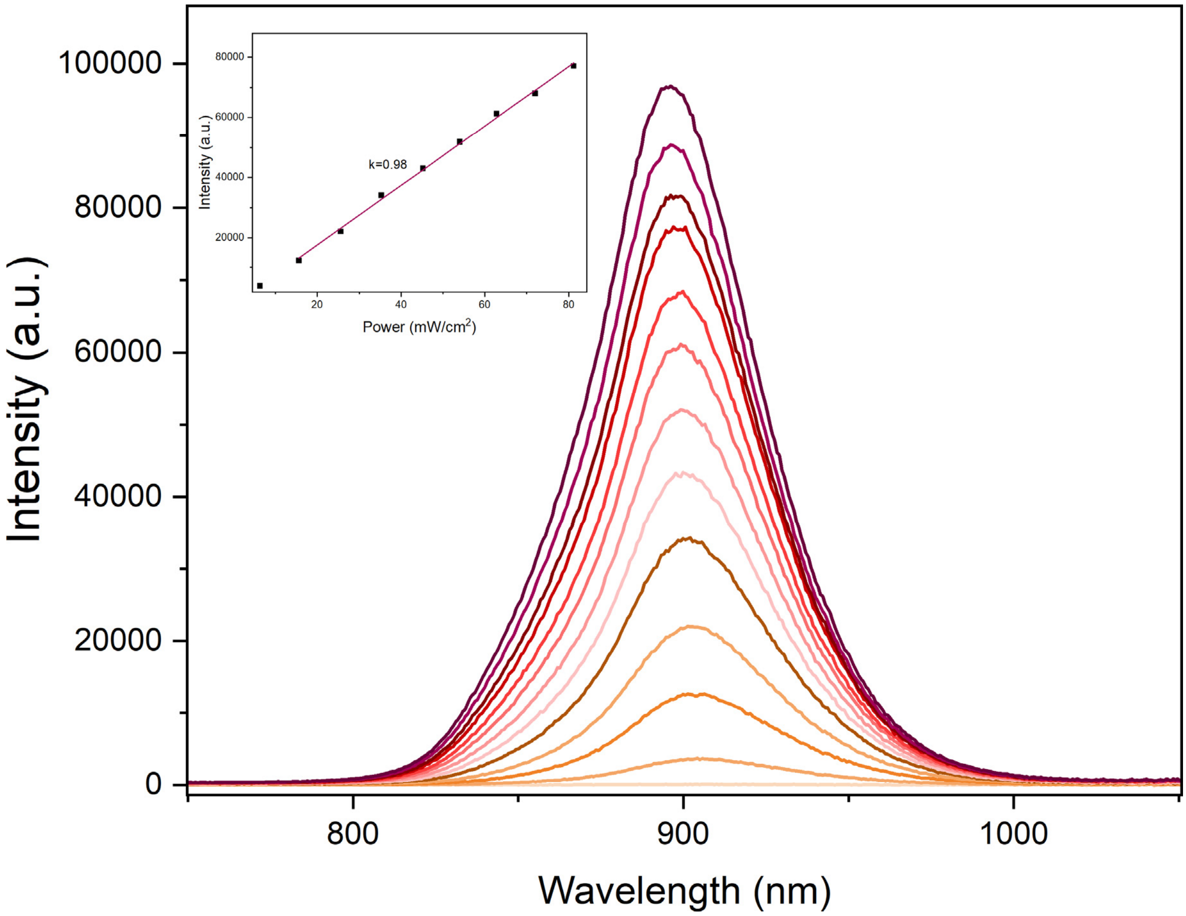

Figure S7. Dependence of PL intensity of $AZPbI_3$ on excitation power.

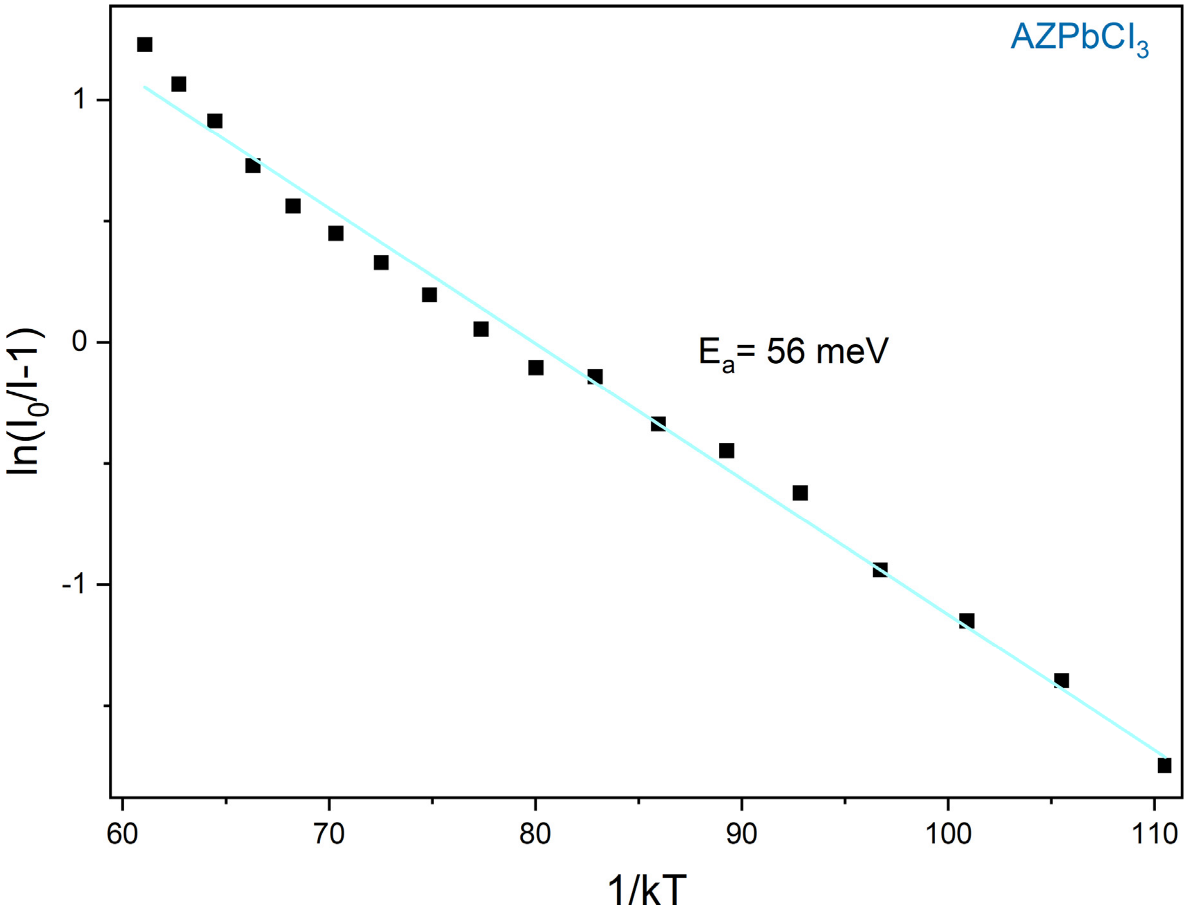

Figure S8. Logarithm of $I_0/I-1$ as a function of $1/k_BT$, where *I*, I_0 and k_B correspond to emission intensity of $AZPbCl_3$ at a given temperature, emission intensity at 80 K, and the Boltzmann constant, respectively. E_a was extracted by fitting to the Arrhenius equation.

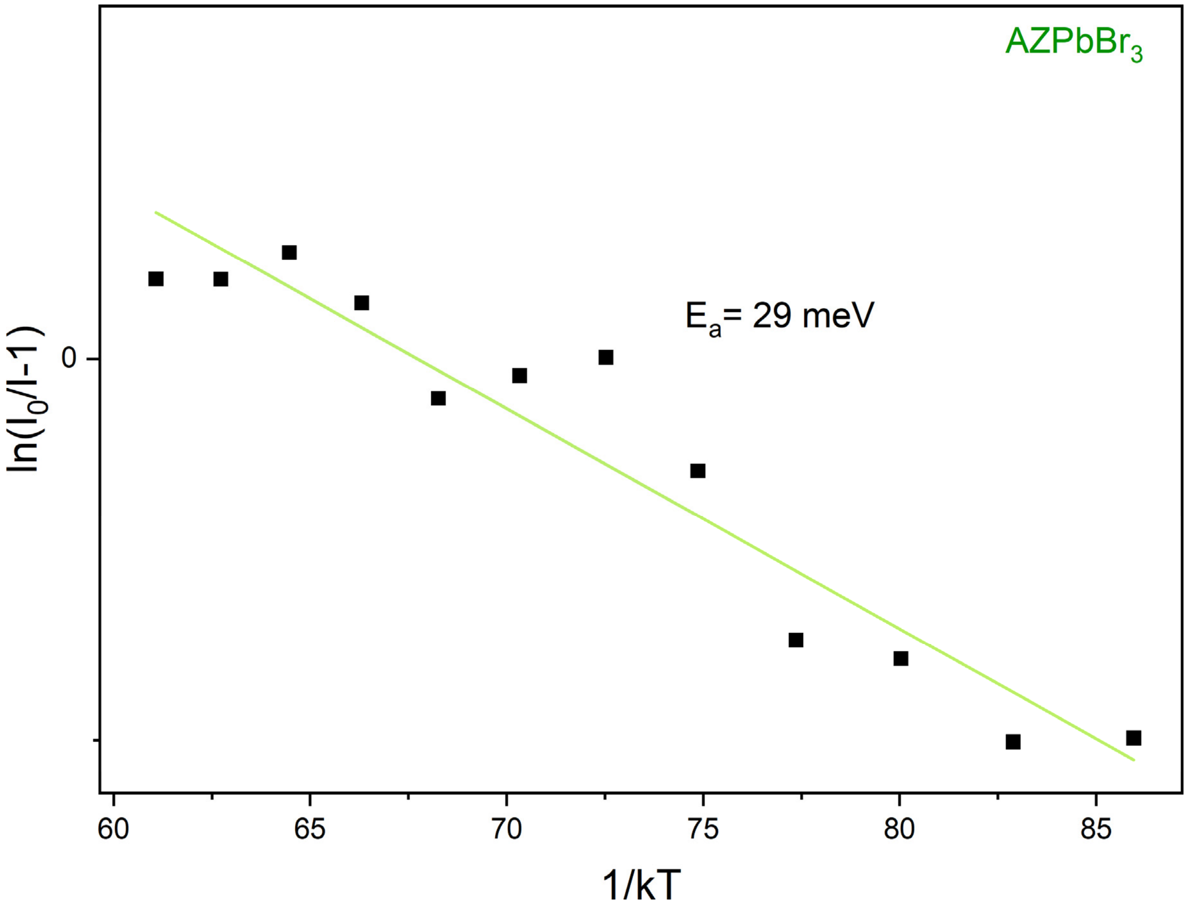

Figure S9. Logarithm of I_0/I-1 as a function of 1/k_BT, where *I*, I_0 and k_B correspond to emission intensity of $AZPbBr_3$ at a given temperature, emission intensity at 80 K, and the Boltzmann constant, respectively. E_a was extracted by fitting to the Arrhenius equation.

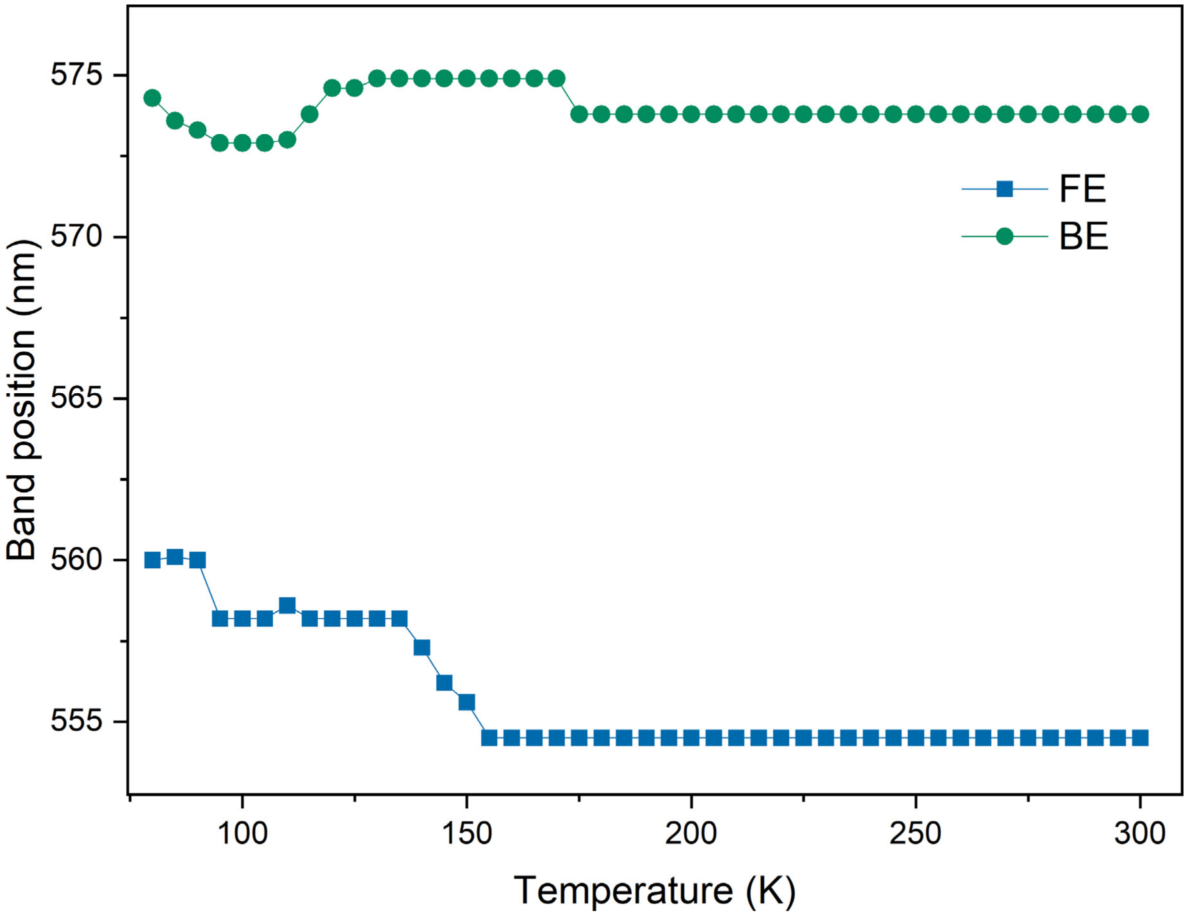

Figure S10. Temperature dependence of band centre positions for FE and BE bands of $AZPbBr_3$.

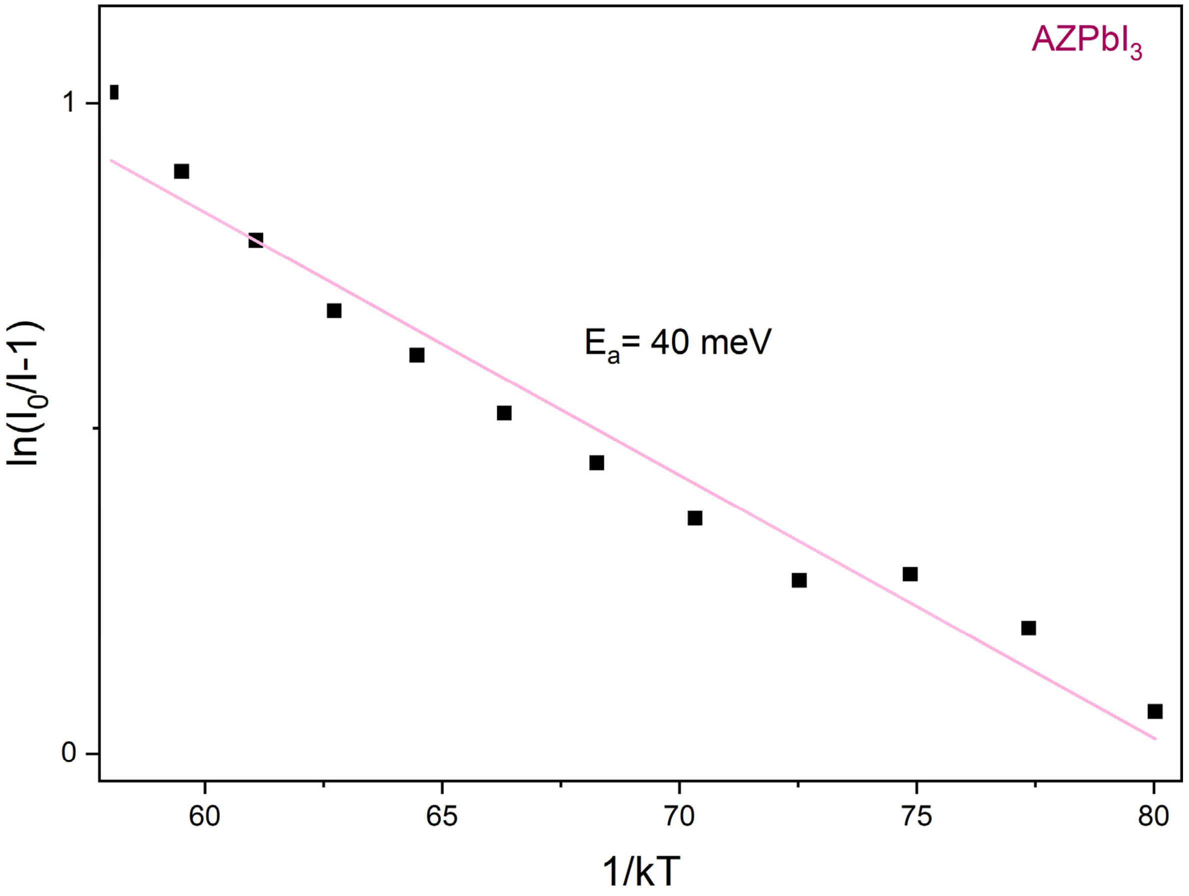

Figure S11. Logarithm of I_0/I-1 as a function of 1/k_BT, where I, I_0 and k_B correspond to emission intensity of $AZPbI_3$ at a given temperature, emission intensity at 80 K, and the Boltzmann constant, respectively. E_a was extracted by fitting to the Arrhenius equation.

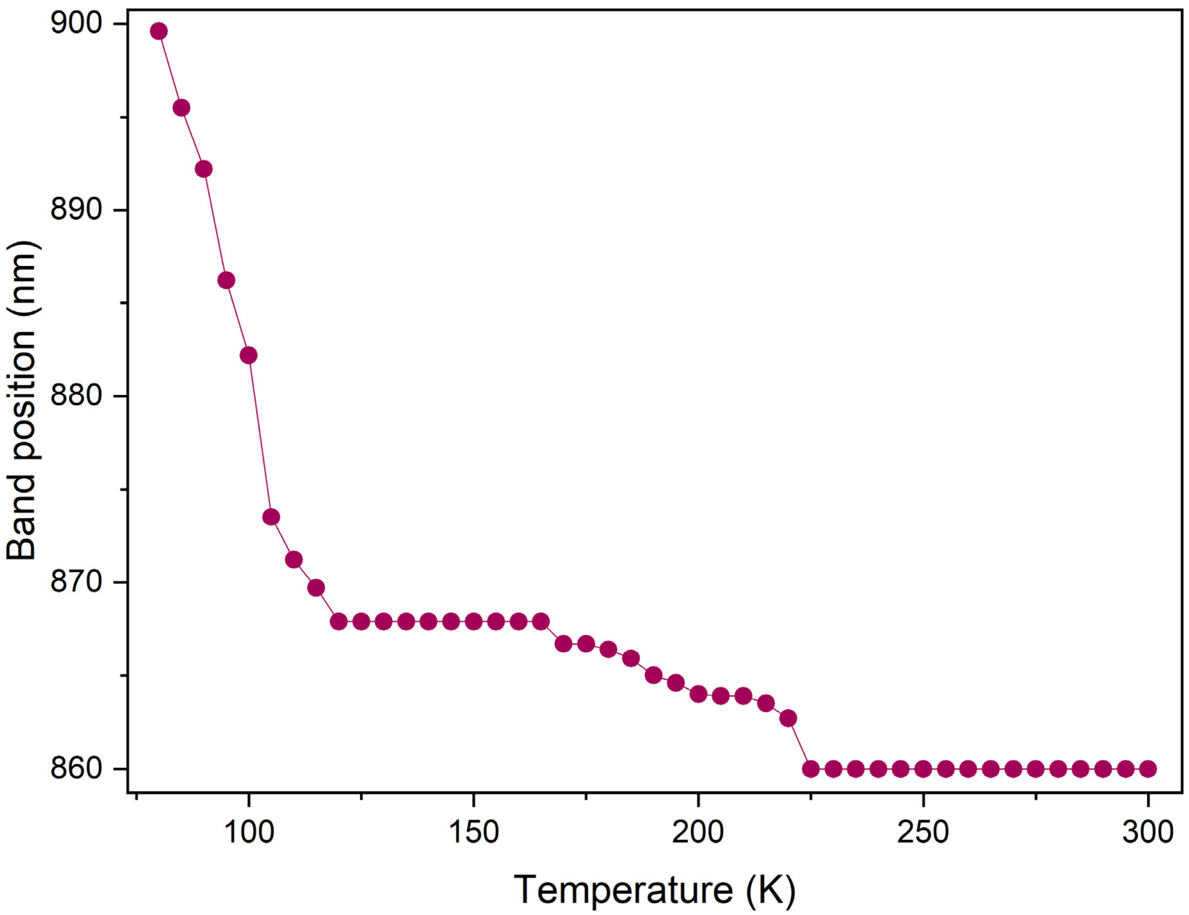

Figure S12. Temperature dependence of band centre positions for FE band of $AZPbI_3$.

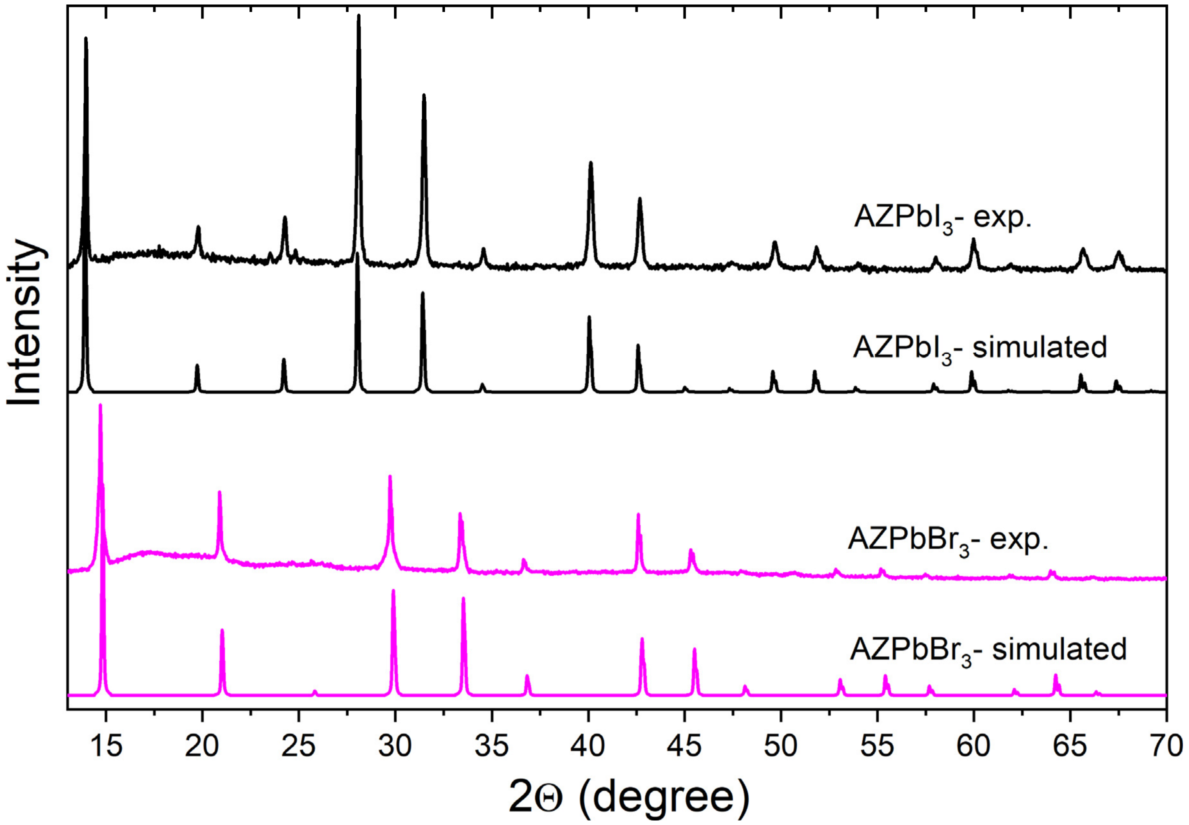

Figure S13. Experimental and simulated powder XRD patterns of $AZPbBr_3$ and $AZPbI_3$.

Table S1. Raman wavenumbers (in cm^{-1}) of $AZPbX_3$ (X=Cl, Br, I) together with the proposed assignment.[a]

$AZPbCl_3$	$AZPbBr_3$	$AZPbI_3$	assignment
3225w,b	3217w,b		$\nu(NH_2)$
	3170w,b	3158w,b	$\nu(NH_2)$
3128w	3121w	3107vw	$\nu_{as}(CH_2)$
3046sh	3035sh	3022w	$\nu_s(CH_2)$
3037m	3029m	3014sh	$\nu_s(CH_2)$
1551w	1547w	1541w	$\delta(NH_2)$
1456w	1453w	1449w	$\delta(CH_2)$
1444sh	1440sh		$\delta(CH_2)$
1232s	1228s	1223s	ring stretch
1219w	1215w	1210w	$\tau(CH_2)$
1139vw	1138vw	1132vw	$\tau(NH_2)$?
1114m	1113m	1111m	$\omega(CH_2)$
1002vw,b	1007vw,b	998w	$\omega(NH_2)$?
926vw	919w	907w	$\rho(NH_2)$?
875m	871m	863m	ring deformation
835sh	827sh	817w	$\rho(CH_2)$
808m	802m	794m	ring deformation
771vw	767vw	761vw	$\rho(CH_2)$
468m	308m	240m	AZ-cage mode
139vs	132vs	122vs	Pb-X stretch

[a] key: vs, very strong; s, strong; m, medium; w, weak; vw, very weak; sh, shoulder; ν, stretching; δ, bending (scissoring); ρ, rocking; ω, wagging; τ, twist; L, librational mode; T', translational mode.
